# The importance of the *UGT1A1* variants in the development of osteopenia and osteoporosis in postmenopausal women

**DOI:** 10.1038/s41598-021-96429-x

**Published:** 2021-08-30

**Authors:** Anna Bogacz, Adam Kamiński, Małgorzata Łochyńska, Izabela Uzar, Jarosław Gorący, Daniel Kotrych, Agnieszka Seremak-Mrozikiewicz, Bogusław Czerny

**Affiliations:** 1 Department of Pharmacology and Phytochemistry, Institute of Natural Fibres and Medicinal Plants, Kolejowa 2, 62-064 Plewiska, Poland; 2 Department of Orthopaedics and Traumatology, Independent Public Clinical Hospital No. 1, Pomeranian Medical University in Szczecin, Unii Lubelskiej 1, 71-252 Szczecin, Poland; 3 Institute of Natural Fibres and Medicinal Plants, Wojska Polskiego 71b, 60-630 Poznan, Poland; 4 Department of Pharmacology and Pharmacoeconomics, Pomeranian Medical University in Szczecin, Żołnierska 48, 71-230 Szczecin, Poland; 5 Independent Laboratory of Invasive Cardiology, Pomeranian Medical University in Szczecin, al. Powstańców Wlkp. 72, 70-111 Szczecin, Poland; 6 Department of Orthopaedics, Traumatology and Orthopaedic Oncology, Pomeranian Medical University in Szczecin, Unii Lubelskiej 1, 71-252 Szczecin, Poland; 7 Division of Perinatology and Women’s Diseases, Poznan University of Medical Sciences, Polna 33, 60-535 Poznan, Poland; 8 Department of Pharmacology and Pharmacoeconomics, Pomeranian Medical University in Szczecin, Żołnierska 48, 71-230 Szczecin, Poland

**Keywords:** Molecular biology, Molecular medicine

## Abstract

The UDP-glucuronosyltransferase 1A1 (UGT1A1) is involved in the process of estrogen conjugation and elimination. The aim of the study was to analyze whether the *UGT1A1* genetic variants are associated with the development of osteopenia and osteoporosis in postmenopausal women. The analysis of the rs4148323 (UGT1A1*6) and rs3064744 (UGT1A1*28) variants in the *UGT1A1* gene was conducted using real-time PCR. A significant correlation was observed between the genotypes of the rs3064744 (UGT1A1*28) sequence variant and body mass in women with osteoporosis. The analysis of the Z-score values revealed that women with osteoporosis and carrying the 6/6 variant had the lowest Z-score values as compared to women with the 6/7 and the 7/7 variants (− 1.966 ± 0.242 vs. − 1.577 ± 0.125 and − 1.839 ± 0.233). In addition, the odds ratio for the investigated genotypes (6/6, 6/7, 7/7) indicated an increased risk for osteopenia and osteoporosis in women with the 7/7 homozygous genotype. The analysis of the frequencies of the GG, GA and AA genotypes of the rs4148323 *UGT1A1* gene showed no statistically significant differences between the groups. Our analysis revealed that the *UGT1A1* rs3064744 variant may affect the risk of developing osteoporosis in postmenopausal Polish women. The *UGT1A1* rs4148323 variant is not directly associated with the development of osteopenia and osteoporosis.

## Introduction

Osteoporosis belongs to the group of ‘diseases of affluence’, with the loss of bone mass and deteriorated bone structure as the dominant symptoms. The pathomechanism of osteoporosis is complex and multifactorial, associated with changes in the concentration profiles of hormones, cytokines, and growth factors. Over 30% of all postmenopausal women are affected by osteoporosis. According to the data from the International Osteoporosis Foundation, 200 million women worldwide are diagnosed with this disease (1/5 at the age of 70 and as many as 2/3 over the age of 90). In addition, 1/3 of the women suffer osteoporosis-related bone fractures, which is a typical occurrence in osteoporosis^1^. Initially, the symptoms of the disease are hardly noticeable by the patient, with low-energy fractures as the first indication of abnormal bone metabolism^2^. For this reason, it is necessary to raise awareness about the risk factors and symptoms of osteoporosis, which in turn will help to minimize the effects of bone mass loss^3^. There are four types of osteoporosis: (1) true osteoporosis, in the course of which normal physical activity causes pain or fractures, mainly of the spine; (2) physiological osteopenia, with lower bone mechanical resistance as the result of low physical activity and decreased muscle strength, and fractures occurring as the consequence of high trauma; (3) combination of true osteoporosis with physiological osteopenia; and (4) transient osteopenia, which is the result of reduced physical activity associated with injury or disease^4^. A better understanding of the molecular mechanisms underlying osteoporosis is vital for the diagnosis and treatment, not to mention the earliest possible identification of the factors predisposing to the development of the disease^5^. So far, numerous molecular analyses were performed to investigate the possible role of the genetic factors in the etiology of osteoporosis^6^. The risk of osteoporosis and osteopenia has been linked to genetic variants, especially the *COL1A1*, *VDR*, *BMP2*, *TLR* genes, as well as the *LRP5* gene involved in the Wnt/β-catenin signaling pathway^7–12^.

The search for new genes which play an important role in the regulation of bone mass and the development of osteoporosis continues. Previous GWAS studies demonstrated a link between polymorphisms of the genes related to estrogen metabolism and osteoporosis and the risk of bone fracture^12^. Recently, much attention has been paid to the potential biomarkers, including UDP-glucuronosyltransferases (UGTs).

UDP-glucuronosyltransferases comprise a superfamily of membrane-bound conjugating enzymes involved in the inactivation and elimination of numerous endogenous and exogenous compounds. UGTs catalyze the glucuronidation reaction, which is associated with the metabolism of bilirubin, bile acids, fatty acids, steroid hormones, thyroid hormones, and fat-soluble vitamins^12,13^. Glucuronidation is also one of the most important phase II biotransformation reactions^14^. UGTs are expressed in various tissues: brain, liver, kidneys, small intestine, colon, stomach, lungs, epithelium, ovaries, testes, mammary glands, and prostate^13–15^. *UGT1A1* is expressed in the uterus and is involved in the conjugation and elimination of estrogens. Studies indicate that permanent estrogen deficiency after menopause is the main cause of osteoporosis in older women^16^. However, the relationship between osteoporosis and the *UGT1A1* gene variant in Caucasian postmenopausal women remains to be fully elucidated. Therefore, researchers are constantly looking for new genetic variants that could affect the risk of developing osteoporosis. Conducted scientific studies analyze the influence of genetic variants of the *UGT1A1*, including UGT1A1*6 (211G>A, rs4148323), UGT1A1*27 (686C>A, rs35350960), UGT1A1*60 (− 3263T>A, rs4124874), and TA repeat variation of UGT1A1*28 (A(TA)_7_TAA, rs3064744) on the risk of developing osteoporosis or other pathological entities, e.g. Gilbert's Syndrome. Moreover, it has been shown that the UGT1A1*28 variant influences glucuronidation of bazedoxifene used for the prevention and treatment of osteoporosis.

The aim of the study was to investigate whether the *UGT1A1* rs3064744 (UGT1A1*28) and the rs4148323 (UGT1A1*6) genetic variants are associated with the development of osteopenia and osteoporosis in postmenopausal women.

## Methods

### Patients

The study included 675 Polish postmenopausal women (109 with osteopenia, 333 with osteoporosis and 233 healthy controls). BMD measurements were performed at the Laboratory of Densitometry, Clinical Hospital No. 1, Pomeranian Medical University in Szczecin. BMD was measured in the lumbar spine, from L2 to L4 vertebrae, using DEXA (Dual Energy X-ray Absorptiometry). Densitometry was performed using the LUNAR DPX 100 camera (Lunar Corp., Madison, USA). Normal BMD value using DEXA is between one standard deviation from the mean in relation to the age of peak bone mass (− 1 < T-score > 1). Based on these measurements, the women were classified into the following groups: osteopenia (− 2.5 < T-score < − 1), osteoporosis (T-score < − 2.5), and normal T-score—controls (T-score > − 1). The ratio of mean BMD in relation to mean value for young adults (YA) and in comparison to age (age-matched, AM), was also evaluated*.* Furthermore, height and weight were measured, and the body mass index (BMI) was calculated. Data on disease manifestation, drug use, age at first and last menstruation, gravidity and parity, and birth weight were collected. The inclusion criteria for the study were as follows: menopause at least 1 year before, no hormone replacement therapy (HRT) or drugs affecting bone mass (selective estrogen receptor modulators SERMs, calcitonin, bisphosphonates, heparin, steroids, thyroid hormones, antiepileptic drugs, GnRH analogues, tibolone). Patients with endocrine and metabolic disorders, hematological diseases, kidney disease, cancers, autoimmune and connective tissue diseases, and after bilateral ovariectomy were excluded from the analysis. Additionally, women who did not smoke were qualified for the study because tobacco smoking may increase the risk of osteoporosis. Moreover, women were not selected in terms of physical activity. The study procedures were approved by the Bioethics Committee of Poznan University of Medical Sciences, Poland (no. 1415/03 (158/06)). The Ethics statement was approved according to Helsinki Declaration. Written informed consent was obtained from all participants.

### Analysis of the rs4148323 (UGT1A1*6) and the rs3064744 (UGT1A1*28) variants in the *UGT1A1* gene

Blood samples were collected at the Department of Orthopedics and Traumatology, Pomeranian Medical University. The analysis of the *UGT1A1* gene variants was conducted at the Department of Stem Cell and Regenerative Medicine, Institute of Natural Fibers and Medicinal Plants, Poznan. Genomic DNA was extracted from peripheral blood using QIAamp Blood Kit (Qiagen GmbH, Hilden, Germany), in accordance with the manufacturer’s protocol. DNA concentration was measured using DeNovix DS-11 Spectrophotometer (DeNovix Inc., USA). LightCycler FastStart DNA Master HybProbe (Roche Diagnostics) assay and LightCycler®480 instrument for the *UGT1A1* gene genotyping were used. Determination of the rs4148323 and the rs3064744 variants of the *UGT1A1* gene was performed using LightSNiP (TIBMolbiol), which contained the primers and probes specific for the amplified fragment. PCR was performed in 10 μl reaction mixture according to the manufacturer's protocol under the following conditions: initial denaturation at 95 °C for 10 min, and 35 cycles as follows: denaturation at 95 °C for 10 s, annealing at 60 °C for 10 s, elongation for 15 s at 72 °C, and melting for 30 s at 95 °C and 40 °C for 120 s. The *UGT1A1* sequence variants were observed as different melting curves of the PCR products. The UGT1A1 promoter region generally contains six TA repeats, but alleles containing seven repeats lead to reduced gene expression (UGT1A1*28 variant, rs3064744). All genotyping data obtained were double-assessed to minimize error. A duplicate plate was entered to check the quality of genotyping. No incompatibilities were observed. Additionally, positive controls for heterozygote, wild-type and mutant homozygote were used.

### Statistical analysis

Data analysis was performed using SPSS Statistics 17.0 for Windows. The observed frequencies were compared with the expected frequencies and tested for the Hardy–Weinberg equilibrium. The expected results are presented with 95% confidence intervals (CI). The odds ratio (OR) for the genotypes and the alleles was calculated. Then, the effect of the *UGT1A1* genetic variants on T-score, Z-score, L2L4AM (bone mineral density compared with an age-matched), L2L4YA (bone mineral density in young adult), L2L4BMD (bone mineral density between lumbar vertebrae L2–L4), BMI (body mass index), and other clinical parameters was evaluated. Correlation analysis between the genotypes and the clinical parameters was conducted using one-way ANOVA. The p-value of < 0.05 was considered as statistically significant.

All methods were carried out in accordance with relevant guidelines and regulations.

## Results

The analysis of the rs4148323 (UGT1A1*6) and the rs3064744 (UGT1A1*28) variants in the *UGT1A1* gene was based on different melting curves of the PCR products. Table 1 presents the clinical parameters of postmenopausal women classified into the groups with osteoporosis, osteopenia and controls. The association of the *UGT1A1* on the risk of developing osteopenia and osteoporosis was evaluated, which was then correlated with the clinical parameters, including bone parameters. Analyzing the obtained results in the women with osteoporosis, we observed that the body mass was lower in carriers of genotypes 6/6 (60.379 ± 1.265 kg) and 6/7 (60.325 ± 1.204 kg) compared to women with genotypes 7/7 (66.833 ± 2.023 kg, p < 0.005). The inverse relationship was observed in the control group (genotype 6/6: 68.045 ± 2.241 kg and genotype 6/7: 70.212 ± 2.228 kg vs. genotype 7/7: 64.571 ± 2.930 kg, p = 0.142) and women with osteopenia (genotype 6/6: 66.438 ± 1.712 kg and genotype 6/7: 64.954 ± 1.801 vs. genotype 7/7: 63.526 ± 1.714, p = 0.243).

**Table 1:** Characteristics of the postmenopausal women with osteopenia, osteoporosis and controls taking part in the study of the UGT1A1*28 genetic variant.

Genotype	6/6	6/7	7/7	
Osteopenia				
	Mean ± SD n = 43	Mean ± SD n = 46	Mean ± SD n = 20	*p*
Age (years)	52.211 ± 7.117	53.727 ± 9.197	53.842 ± 6.702	0.543
T-score	− 1.828 ± 0.706	− 1.841 ± 0.065	− 1.720 ± 0.097	0.425
Z-score	− 0.921 ± 0.114	− 0.808 ± 0.118	− 0.816 ± 0.227	0.354
Body mass (kg)	66.438 ± 1.712	64.954 ± 1.801	63.526 ± 1.714	0.243
BMI (kg/m^2^)	24.665 ± 0.581	24.553 ± 0.701	24.357 ± 0.684	0.455
Birth weight (g)	3169.166 ± 106.546	3306.667 ± 90.707	3356.032 ± 319.352	0.365
Years of reproduction	35.791 ± 5.021	36.727 ± 4.311	36.300 ± 6.766	0.244
Age of first menstruation	12.791 ± 2.734	13.045 ± 2.126	13.500 ± 2.368	0.436
Age of last menstruation	48.500 ± 4.338	50.103 ± 4.512	49.571 ± 5.139	0.432
Years after menopause	6.500 ± 4.428	7.810 ± 6.214	6.400 ± 7.411	0.542
BMD L2–L4 (g/cm^2^)	0.947 ± 0.054	0.964 ± 0.028	1.016 ± 0.031	0.632
BMD L2–L4 YA (%)	76.838 ± 2.324	83.325 ± 2.510	80.166 ± 7.211	0.268
BMD L2–L4 AM (%)	86.720 ± 2.042	89.194 ± 2.931	92.823 ± 2.753	0.366

Osteoporosis				
	Mean ± SD n = 129	Mean ± SD n = 154	Mean ± SD n = 50	*p*
Age (years)	56.227 ± 8.189	56.857 ± 8.256	57.222 ± 11.074	0.542
T-score	− 3.319 ± 0.097	− 3.081 ± 0.074	− 3.223 ± 0.219	0.326
Z-score	− 1.966 ± 0.242	− 1.577 ± 0.125	− 1.839 ± 0.233	0.096
Body mass (kg)*	60.379 ± 1.265	60.325 ± 1.204	66.833 ± 2.023	0.045
BMI (kg/m^2^)	23.407 ± 0.551	23.567 ± 0.479	25.538 ± 0.570	0.243
Birth weight (g)	3233.333 ± 231.142	3155.714 ± 227.165	3050.012 ± 354.101	0.344
Years of reproduction	34.900 ± 5.379	35.575 ± 5.131	36.909 ± 4.276	0.348
Age of first menstruation	12.650 ± 2.580	13.393 ± 2.014	11.905 ± 1.375	0.632
Age of last menstruation	46.111 ± 5.514	49.052 ± 4.645	48.667 ± 4.313	0.472
Years after menopause	12.100 ± 4.428	9.666 ± 5.374	11.454 ± 4.612	0.266
BMD L2–L4 (g/cm^2^)	0.985 ± 0.023	0.972 ± 0.028	0.983 ± 0.032	0.282
BMD L2–L4 YA (%)	82.184 ± 1.927	81.065 ± 2.324	81.933 ± 2.708	0.362
BMD L2–L4 AM (%)	90.315 ± 2.007	89.239 ± 2.141	90.133 ± 3.421	0.423

Controls				
	Mean ± SD n = 88	Mean ± SD n = 120	Mean ± SD n = 25	*p*
Age (years)	54.632 ± 5.041	53.247 ± 8.736	52.428 ± 13.044	0.442
T-score	0.045 ± 0.234	0.149 ± 0.161	− 0.002 ± 0.371	0.096
Z-score	0.687 ± 0.158	0.782 ± 0.277	0.042 ± 0.343	0.064
Body mass (kg)	68.045 ± 2.241	70.212 ± 2.228	64.571 ± 2.930	0.142
BMI (kg/m^2^)	25.741 ± 0.887	26.431 ± 0.947	25.139 ± 1.217	0.352
Birth weight (g)	3587.272 ± 55.176	3750.546 ± 343.016	3495.500 ± 66.272	0.426
Years of reproduction	35.810 ± 5.221	36.416 ± 5.122	38.509 ± 6.256	0.466
Age of first menstruation	13.550 ± 1.780	13.323 ± 1.816	13.255 ± 2.775	0.482
Age of last menstruation	49.151 ± 3.541	50.832 ± 4.725	52.627 ± 4.114	0.375
Years after menopause	6.562 ± 4.026	7.756 ± 5.424	6.724 ± 9.512	0.421
BMD L2–L4 (g/cm^2^)	0.986 ± 0.029	0.982 ± 0.035	1.101 ± 0.081	0.244
BMD L2–L4 YA (%)	82.142 ± 3.045	78.925 ± 2.904	91.091 ± 4.269	0.076
BMD L2–L4 AM (%)	89.071 ± 2.004	89.703 ± 3.018	97.601 ± 7.440	0.088

*BMI* body mass index, *BMD L2–L4 YA* bone mineral density in young adult, *BMD L2–L4 AM* bone mineral density compared with an age-matched, *6/6* the UGT1A1 promotor region contains six TA repeats, *7/7* the UGT1A1 promotor region contains seven TA repeats.

Interestingly, women with osteopenia and osteoporosis had lower birth weight as compared to the control group. Analyzing the Z-score values, we also determined that women with osteoporosis and carrying the 6/6 variant had the lowest Z-score value as compared to women with the 6/7 and 7/7 variants (− 1.966 ± 0.242 vs*.* − 1.577 ± 0.125 and − 1.839 ± 0.233, p = 0.096). For the T-score values in relation to the genotypes for the UGT1A1 variant, no differences were observed between the studied groups. In addition, the effect of the *UGT1A1* genetic variants on the duration of a woman’s reproductive years was analyzed. No statistically significant differences among the groups were found, because the reproductive years of an average woman were between the ages of 12 and 52 in all groups*.* The frequency of homozygous 6/6 genotype of the UTG1A1*28 variant (rs3064744) did not differ in the group of women with osteopenia and postmenopausal controls (Table 2). A slightly higher 6/7 genotype frequency was demonstrated in the control group, whereas the frequency of the 7/7 genotype was higher in the group with osteopenia as compared to controls (Table 2). In addition, heterozygous 6/7 genotype frequency was slightly lower in the group of women with osteoporosis as compared to controls (46.2% vs. 51.5%, p = 0.146, OR = 0.81, 95% CI 0.57–1.15) (Table 3). A higher frequency of the 7/7 genotype was observed in the osteoporosis group as well as osteopenia as compared to controls (15.0% vs. 10.7%, p = 0.146, OR = 1.47, 95% CI 0.86–2.56; 18.3% vs. 10.7%, p = 0.049, OR = 1.87, 95% CI 0.93–3.70, respectively). In addition, the odds ratio for the investigated genotypes (6/6, 6/7, 7/7) indicated a higher risk for osteopenia and osteoporosis in women with the 7/7 homozygous genotype (Tables 2 and 3).

**Table 2:** The frequency of the specific alleles and genotypes of the UGT1A1*28 variant in the group of women with osteopenia and controls.

Genotypes	Osteopenia		Control		OR	95% CI	p
	Observed value n (%)	Expected value %	Observed value n (%)	Expected value (%)			
6/6	43 (39.5)	36.7	88 (37.8)	40.3	1.07	0.65–1.75	0.049
6/7	46 (42.2)	47.8	120 (51.5)	46.4	0.69	0.42–1.12	
7/7	20 (18.3)	15.5	25 (10.7)	13.3	1.87	0.93–3.70	
Total	109 (100%)	100.00	233 (100%)	100.00	–	–	–

The expected value was calculated in accordance with the Hardy–Weinberg equilibrium (HWE). HWE equilibrium test was used to obtain the exact *p* value.

*OR* odds ratio.

**Table 3:** The frequency of specific alleles and genotypes of the UGT1A1*28 variant in the group of women with osteoporosis and controls.

Genotypes	Osteoporosis		Control		OR	95% CI	p
	Observed value n (%)	Expected value (%)	Observed value n (%)	Expected value (%)			
6/6	129 (38.7)	38.3	88 (37.8)	40.3	1.04	0.73–1.49	0.146
6/7	154 (46.2)	47.2	120 (51.5)	46.4	0.81	0.57–1.15	
7/7	50 (15.0)	14.5	25 (10.7)	13.3	1.47	0.86–2.56	
Total	333 (100%)	100.00	233 (100%)	100.00	–	–	–

The expected value was calculated in accordance with the Hardy–Weinberg equilibrium (HWE). HWE equilibrium test was used to obtain the exact *p* value.

*OR* odds ratio.

The analysis of the frequencies of the GG, GA and AA genotypes in the rs4148323 polymorphism of the *UGT1A1* gene showed no statistically significant differences between the investigated groups (Table 4). The GG genotype was dominant among the women with osteopenia, osteoporosis and controls, while the GA genotype was sporadic in the control group and women with osteopenia. The AA genotype was not found in any of the groups. As far as frequency of the rs4148323 polymorphism and the clinical parameters were concerned, no statistically significant differences were observed (Table 4).

**Table 4:** Characteristics of the postmenopausal women with osteopenia, osteoporosis and normal T-score taking part in the study of the rs4148323 genetic variant of *UGT1A1* gene.

Genotype	GG	GA	AA
Osteopenia			
	Mean ± SD n = 108	Mean ± SD n = 1	Mean ± SD n = 0
Age (years)	53.332 ± 8.17	52	–
T-score	− 1.803 ± 0.439	− 1.900	–
Z-score	− 0.929 ± 0.112	− 0.487	–
Body mass (kg)	65.338 ± 1.071	74.000	–
BMI (kg/m^2^)	24.647 ± 0.384	27.180	–
Birthweight (g)	3346.543 ± 100.622	3200.000	–
Years of reproduction	35.643 ± 4.043	36	–
Age of first menstruation	12.432 ± 4.321	11	–
Age of last menstruation	48.435 ± 4.133	47	–
Years after menopause	6.732 ± 4.221	5	–
BMD L2–L4 (g/cm^2^)	0.963 ± 0.022	0.964	–
BMD L2–L4 YA (%)	80.582 ± 1.932	80.000	–
BMD L2–L4 AM (%)	88.531 ± 2.014	95.000	–

Osteoporosis			
	Mean ± SD n = 333	Mean ± SD n = 0	Mean ± SD n = 0
Age (years)	54.447 ± 4.17	–	–
T-score	− 3.164 ± 0.056	–	–
Z-score	− 3.569 ± 1.946	–	–
Body mass (kg)	61.208 ± 0.938	–	–
BMI (kg/m^2^)	23.787 ± 0.318	–	–
Birth weight (g)	3141.250 ± 134.079	–	–
Years of reproduction	35.993 ± 4.023	–	–
Age of first menstruation	13.092 ± 4.317	–	–
Age of last menstruation	48.981 ± 4.032	–	–
Years after menopause	8.572 ± 4.208	–	–
BMD L2–L4 (g/cm^2^)	0.975 ± 0.014	–	–
BMD L2–L4 YA (%)	81.278 ± 1.240	–	–
BMD L2–L4 AM (%)	89.504 ± 1.123	–	-

Controls			
	Mean ± SD n = 231	Mean ± SD n = 2	Mean ± SD n = 0
Age (years)	53.617 ± 8.271	55.037 ± 2.831	
T-score	0.110 ± 0.120	− 0.795 ± 0.095	–
Z-score	0.689 ± 0.205	− 0.055 ± 0.475	–
Body mass (kg)	68.633 ± 1.571	74.052 ± 10.211	–
BMI (kg/m^2^)	26.031 ± 0.591	26.345 ± 1.65	–
Birth weight (g)	3630.556 ± 116.258	3652.500 ± 164.026	–
Years of reproduction	36.373 ± 5.523	37.563 ± 2.433	–
Age of first menstruation	13.522 ± 1.812	11.542 ± 0.717	–
Age of last menstruation	50.441 ± 4.332	49.000 ± 1.412	–
Years after menopause	7.112 ± 5.728	6.040 ± 1.412	–
BMD L2–L4 (g/cm^2^)	0.973 ± 0.023	0.955 ± 0.025	–
BMD L2–L4 YA (%)	81.333 ± 1.291	80.703 ± 1.104	–
BMD L2–L4 AM (%)	87.420 ± 2.072	88.325 ± 2.475	–

*BMI* body mass index, *BMD L2–L4 YA* bone mineral density in young adult, *BMD L2–L4 AM* bone mineral density compared with an age-matched.

The AA genotype for the rs4148323 variant in the UGT1A1 gene was not identified. The GA genotype for women with osteoporosis was also not identified.

## Discussion

In this study, the *UGT1A1* genetic variant (UGT1A1*28) was used as a complementary marker of bone mass loss. Early identification and detection of the factors predisposing to the development of osteopenia or osteoporosis allow to implement appropriate prophylaxis and, if necessary, initiate pharmacotherapy. Changes in the parameters such as bone density and bone mass affect predominantly postmenopausal women^1^, which is the consequence of the changes in their hormonal profile. In postmenopausal women, estrogens are synthesized almost exclusively from the androstenedione formed in the adrenal glands, which is converted into estrone in extraglandular tissues^17^. With the increase in body weight and fat content, which is observed in postmenopausal women, the number of estrogen sources increases, while bone turnover decreases, with simultaneous increase in bone mass loss, which seems to be the dominant mechanism of bone tissue changes^18–23^.

*UGT1A1* is involved in the process of estrogen conjugation and elimination^16^. In the present study, the frequency of the UGT1A1*28 variant among Caucasian women was assessed. The search for a connection and a possible correlation between the variants of the analyzed gene and various diseases has so far been reported in the literature for neonatal jaundice^24^, and tumors^25,26^, among others. In this study, a comparison of the homozygous 6/6 genotype frequency of the UGT1A1*28 variant (rs3064744) between the women with osteopenia and postmenopausal controls revealed no differences. However, the frequency of the 6/7 genotype was higher in the control group, while the 7/7 genotype seemed to be more common in people with osteopenia and osteoporosis as compared to controls. In addition, the heterozygous 6/7 genotype was found to be slightly less common in women with osteoporosis. The frequencies of the GG, GA and AA genotypes were also analyzed, but no statistically significant differences between the groups were found. Nevertheless, it can be concluded that among the three analyzed genotypes, the GG genotype was dominant, and the AA genotype was not found.

When comparing the frequency of the analyzed genetic variants and the clinical parameters, a correlation between the genotypes of UGT1A1*28 and body mass was observed in the group of women with osteoporosis. No statistically significant differences were found for other clinical parameters. It seems, therefore, that the limited number of parameters between which correlation was found is a favorable phenomenon in the context of the diagnostic process and the use of research on the genetic variants on the development of osteoporosis. It eliminates the need to search for connections with other clinical parameters and, consequently, allows for a more accurate prediction of the actual impact of the polymorphisms on the development of osteoporosis.

The race of the study population is a vital issue in the analysis of genetic variants in terms of their frequency^27–29^. Since the UGT1A1*28 allele occurs mainly in Caucasian and African American^28,29^ populations, while the UGT1A1*6 allele is widely described in Asian^30,31^ populations, taking into account the race parameter seems to be well-justified. The study of the *UGT1A1* variants is not only important in the context of the metabolism of anticancer drugs, but also, bearing in mind the hormonal associations with osteoporosis, because it seems that the UGT1A1*28 genetic variant may affect the rate of estrogen metabolism^32^. Thus, changes in the nucleotide sequence of the *UGT1A1* gene might affect the severity and progression rate of osteoporosis. It is also possible that the described genetic variants may be related to the rate of bone mass loss, thereby affecting the rate of symptom onset. Our results showed the *UGT1A1* rs3064744 (UGT1A1*28) genetic variant may affect the risk of developing osteopenia and osteoporosis in postmenopausal women, especially in the presence of homozygous genotypes containing two mutated alleles. Studies by Trontelj et al. showed that patients with the UGT1A1*28 genetic variant may affect bone mineral density in women with osteoporosis taking raloxifene. They indicated that women with the *28/*28 (7/7) genotype had an increased BMD compared to patients with the *1/*1 (6/6) and *1/*28 (6/7) genotypes^33^.

Our findings regarding lack of an association between the UGT1A1*6 variant with osteoporosis are consistent with the observations made in the population of postmenopausal Japanese women. Yokota et al., also did not report a correlation between the *UGT1A1* variant and osteoporosis^16^. Nevertheless, it should be taken into account that the absence of statistically significant differences between the compared groups may have resulted from the cross-sectional nature of the study. Therefore, when analyzing all persons included in a given group as a whole, statistical significance may not be observed, however, individual variability should not be forgotten^32,34^. In addition, the results obtained in this study revealed a statistically significant correlation between the analyzed genotypes and body weight. Lower body mass was observed in women with osteoporosis as compared to postmenopausal controls. Low body weight is a predisposing factor for developing osteoporosis, although it remains debatable whether obesity can be a protective factor against bone mass loss^35^, although higher body weight in healthy controls may support the hypothesis. Moreover, taking into account the function of the UGT1A1 protein, one of the main proteins involved in glucuronidation of drugs and other compounds^14^, as well as elimination of estrogens and the consequent reduction of their circulating pool^16^, lower weight may be expected in women with osteoporosis as compared to their postmenopausal healthy peers. The observed values of body mass parameters may be related to the fact that an increase in body weight is accompanied by a corresponding increase in insulin resistance, which attempts to be compensated by elevated secretion of insulin, whose receptors are located on the surface of the osteoblasts. In addition, in women with insulin resistance, increased production of the ovarian hormones and a decreased concentration of sex hormone-binding proteins are observed, which translates into enhanced bioavailability of the estrogen pool, which in turn increase bone mass^36,37^. Therefore, it can be assumed that the UGT1A1*28 genetic variant may be related to the transcriptional activity of the gene followed by the level of protein expression. People with the 6/7 genotype are characterized by a 1/3 reduction in UDP-glucuronosyltransferase activity^38^. Molecular analysis performed in this study also showed that the heterozygous 6/7 genotype of the UGT1A1*28 variant was slightly less common in women with osteoporosis (46.2%) as compared to healthy controls (51.5%). It was also observed that the 7/7 genotype was more common in women with osteoporosis and osteopenia as compared to the control group (15.0% vs. 10.7%, p = 0.146, OR = 1.47, 95% CI 0.86–2.56; 18.3% vs. 10.7%, p = 0.049, OR = 1.87, 95% CI 0.93–3.70, respectively). Hence, it seems safe to conclude that, as far as the Polish population is concerned, low frequency of the 6/7 genotype and high frequency of the 7/7 genotype are characteristic for pathological conditions, e.g., Gilbert's Syndrome, osteopenia, osteoporosis^39^. Molecular analysis of osteoporosis is one of the most dynamically developing areas of research related to bone biology. Therefore, studies focusing on the analysis of genetic variants of the "candidate genes" to be used as complementary molecular markers of bone mass disorders are constantly gaining importance.

In conclusion, the results of the present study indicate that the *UGT1A1* rs3064744 (UGT1A1*28) genetic variant may affect the risk of developing osteopenia and osteoporosis in postmenopausal women, especially in the presence of homozygous genotypes containing two mutated alleles. The analysis of the frequencies of the GG, GA and AA genotypes of the rs4148323 *UGT1A1* gene showed no statistically significant differences between the groups. The *UGT1A1* rs4148323 (UGT1A1*6) genetic variant is not directly associated with the development of osteopenia and osteoporosis in postmenopausal Polish women.

## References

[1] MuÑoz M, Robinson K, Shibli-Rahhal A (2020). Bone health and osteoporosis prevention and treatment. Clin. Obstet. Gynecol..

[2] Lorenc R (2017). Guidelines for the diagnosis and management of osteoporosis in Poland: Update 2017. Endokrynol. Pol..

[3] Johnston CB, Dagar M (2020). Osteoporosis in older adults. Med. Clin. N. Am..

[4] Pothiwala P, Evans EM, Chapman-Novakofski KM (2006). Ethnic variation in risk for osteoporosis among women: a review of biological and behavioral factors. J. Womens Health..

[5] Minkin MJ (2019). Menopause: Hormones, lifestyle, and optimizing aging. Obstet. Gynecol. Clin. N. Am..

[6] Corrado A, Cici D, Rotondo C, Maruotti N, Cantatore FP (2020). Molecular basis of bone aging. Int. J. Mol. Sci..

[7] Wolski H (2015). Polymorphism of bone morphogenetic protein (BMP2) and osteoporosis etiology. Ginekol. Pol..

[8] Kaleta B (2015). Toll-like receptor 4 gene polymorphism C1196T in Polish women with postmenopausal osteoporosis—Preliminary investigation. Adv. Clin. Exp. Med..

[9] Wolski H, Drwęska-Matelska N, Seremak-Mrozikiewicz A (2015). The role of Wnt/β-catenin pathway and LRP5 protein in metabolism of bone tissue and osteoporosis etiology. Ginekol. Pol..

[10] Jin H, Evangelou E, Ioannidis JP, Ralston SH (2011). Polymorphisms in the 5′ flank of COL1A1 gene and osteoporosis: Meta-analysis of published studies. Osteoporos. Int..

[11] Mohammadi Z (2014). Association between vitamin D receptor gene polymorphisms (Fok1 and Bsm1) and osteoporosis: A systematic review. J. Diabetes Metab. Disord..

[12] Miners JO, McKinnon RA, Mackenzie PI (2002). Genetic polymorphisms of UDP-glucuronosyltransferases and their functional significance. Toxicology.

[13] Rowland A, Miners JO, Mackenzie PI (2013). The UDP-glucuronosyltransferases: Their role in drug metabolism and detoxification. Int. J. Biochem. Cell Biol..

[14] Guillemette C (2003). Pharmacogenomics of human UDP-glucuronosyltransferase enzymes. Pharmacogenom. J..

[15] Court MH (2012). Quantitative distribution of mRNAs encoding the 19 human UDP-glucuronosyltransferase enzymes in 26 adult and 3 fetal tissues. Xenobiotica.

[16] Yokota M (2015). Polymorphisms of estrogen metabolism-related genes ESR1, UGT2B17, and UGT1A1 are not associated with osteoporosis in surgically menopausal Japanese women. Prz. Menopauzalny..

[17] Ostrowska Z (2009). Menopause, obesity, and bone status. Adv. Hyg. Exp. Med..

[18] Cifuentes M (2003). Bone turnover and body weight relationship differ in normal-weight compared with heavier postmenopausal women. Osteoporos. Int..

[19] Silva HG, Mendonça LM, Conceição FL, Zahar SE, Farias ML (2007). Influence of obesity on bone density in postmenopausal women. Arq. Bras. Endocrinol. Metabol..

[20] Dawson-Hughes B, Shipp C, Sadowski L, Dallal G (1987). Bone density of the radius, spine, and hip in relation to percent of ideal body weight in postmenopausal women. Calcif. Tissue Int..

[21] Nagant de Deuxchaisnes C, Devogelaer JP (1986). Endocrinological status of postmenopausal osteoporosis. Clin. Rheum. Dis..

[22] Papakitsou EF (2004). Body mass index (BMI) and parameters of bone formation and resorption in postmenopausal women. Maturitas.

[23] Reid IR (2006). Obesity and osteoporosis. Ann. Endocrinol..

[24] Liu W (2017). Correlation between UGT1A1 polymorphism and neonatal hyperbilirubinemia of neonates in Wuhan. J. Huazhong Univ. Sci. Technol. Med. Sci..

[25] Peng H, Duan Z, Pan D, Wen J, Wei X (2017). UGT1A1 gene polymorphism predicts irinotecan-induced severe neutropenia and diarrhea in Chinese cancer patients. Clin. Lab..

[26] Jannin A, Hennart B, Adenis A, Chauffert B, Penel N (2017). Life-threatening irinotecan-induced toxicity in an adult patient with alveolar rhabdomyosarcoma: The role of a UGT1A1 polymorphism. Case Rep. Oncol. Med..

[27] Fujita K, Sparreboom A (2010). Pharmacogenetics of irinotecan disposition and toxicity: A review. Curr. Clin. Pharmacol..

[28] Beutler E, Gelbart T, Demina A (1998). Racial variability in the UDP-glucuronosyltransferase 1 (UGT1A1) promoter: A balanced polymorphism for regulation of bilirubin metabolism?. Proc. Natl. Acad. Sci. USA.

[29] Hall D, Ybazeta G, Destro-Bisol G, Petzl-Erler ML, Di Rienzo A (1999). Variability at the uridine diphosphate glucuronosyltransferase 1A1 promoter in human populations and primates. Pharmacogenetics.

[30] Akaba K (1998). Neonatal hyperbilirubinemia and mutation of the bilirubin uridine diphosphate-glucuronosyltransferase gene: A common missense mutation among Japanese, Koreans and Chinese. Biochem. Mol. Biol. Int..

[31] Araki K (2006). Pharmacogenetic impact of polymorphisms in the coding region of the UGT1A1 gene on SN-38 glucuronidation in Japanese patients with cancer. Cancer Sci..

[32] Mróz A, Mazerska Z (2015). Glucuronidation of antitumour therapeutics—Detoxification, mechanism of resistance or prodrug formation?. Adv. Hyg. Exp. Med..

[33] Trontelj J, Marc J, Zavratnik A, Bogataj M, Mrhar A (2009). Effects of UGT1A1*28 polymorphism on raloxifene pharmacokinetics and pharmacodynamics. Br. J. Clin. Pharmcol..

[34] Niemira M, Wiśniewska A, Mazerska Z (2009). Polymorphism and the level of P450 gene xpression in xenobiotic metabolism. Postepy. Biochem..

[35] Noworyta-Ziętara M, Miazgowski T, Krzyżanowska-Świniarska B, Ogonowski J (2008). Does obesity protect against osteoporosis?. Endokrynologia Otyłość i Zaburzenia Przemiany Materii..

[36] Reid IR (2008). Relationships between fat and bone. Osteoporos. Int..

[37] Ahmed LA, Schirmer H, Berntsen GK, Fønnebø V, Joakimsen RM (2006). Features of the metabolic syndrome and the risk of non-vertebral fractures: The Tromsø study. Osteoporos. Int..

[38] Bosma PJ (1995). The genetic basis of the reduced expression of bilirubin UDP-glucuronosyltransferase 1 in Gilbert's syndrome. N. Engl. J. Med..

[39] Moczulski D, Trombik M, Gawlik B, Górczyńska-Kosiorz S, Grzeszczak W (2007). Sensitivity of genetic testing for Gilbert syndrome in Polish population. Gastroenterol. Rev..

